# Glutathione Enhances Antibiotic Efficiency and Effectiveness of DNase I in Disrupting *Pseudomonas aeruginosa* Biofilms While Also Inhibiting Pyocyanin Activity, Thus Facilitating Restoration of Cell Enzymatic Activity, Confluence and Viability

**DOI:** 10.3389/fmicb.2017.02429

**Published:** 2017-12-14

**Authors:** Theerthankar Das, Martin Simone, Amaye I. Ibugo, Paul K. Witting, Mike Manefield, Jim Manos

**Affiliations:** ^1^Department of Infectious Diseases and Immunology, Sydney Medical School, University of Sydney, Sydney, NSW, Australia; ^2^School of Biotechnology and Biomolecular Sciences, University of New South Wales, Sydney, NSW, Australia; ^3^Discipline of Pathology, Sydney Medical School, University of Sydney, Sydney, NSW, Australia

**Keywords:** glutathione, pyocyanin, oxidative stress, biofilm, *P. aeruginosa*, lung epithelial cells

## Abstract

Pyocyanin secreted by *Pseudomonas aeruginosa* is a virulence factor that damages epithelial cells during infection through the action of reactive oxygen species, however, little is known about its direct effect on biofilms. We demonstrated that pyocyanin-producing *P. aeruginosa* strains (PA14WT, DKN370, AES-1R, and AES-2) formed robust biofilms in contrast to the poorly formed biofilms of the pyocyanin mutant PA14Δ*phzA-G* and the low pyocyanin producer AES-1M. Addition of DNase I and reduced glutathione (GSH) significantly reduced biofilm biomass of pyocyanin-producing strains (*P* < 0.05) compared to non-pyocyanin producers. Subsequently we showed that a combined treatment comprising: GSH + DNase I + antibiotic, disrupted and reduced biofilm biomass up to 90% in cystic fibrosis isolates AES-1R, AES-2, LESB58, and LES431 and promoted lung epithelial cell (A549) recovery and growth. We also showed that exogenously added GSH restored A549 epithelial cell glutathione reductase activity in the presence of pyocyanin through recycling of GSSG to GSH and consequently increased total intracellular GSH levels, inhibiting oxidative stress, and facilitating cell growth and confluence. These outcomes indicate that GSH has multiple roles in facilitating a return to normal epithelial cell growth after insult by pyocyanin. With increased antibiotic resistance in many bacterial species, there is an urgency to establish novel antimicrobial agents. GSH is able to rapidly and comprehensively destroy *P. aeruginosa* associated biofilms while at a same time assisting in the recovery of host cells and re-growth of damaged tissue.

## Introduction

The Gram-negative opportunistic bacterium *Pseudomonas aeruginosa* is responsible for chronic and persistent infections in animals and humans and can employ a wide range of virulence factors to maintain infection. In patients with cystic fibrosis (CF), *P. aeruginosa* is the dominant species in CF lung by adolescence, and leads to morbidity and mortality of about 80% of CF patients worldwide (Hoiby, 2011). Studies indicate that infections due to *P. aeruginosa* are more persistent in adult CF patients compared to children and infants (Cox et al., 2010). *P. aeruginosa* associated infections are also a leading cause of airway infections in bronchiectasis, infections of burns and wounds, HIV patients, eye infections due to contact lens contamination and hospital acquired infections in immunocompromised individuals (Gellatly and Hancock, 2013).

As with many pathogenic bacteria, *P. aeruginosa* form structurally integrated biofilms on host surfaces after colonization (Bjarnsholt et al., 2010). Biofilm formation in *P. aeruginosa* is mediated through a complex quorum sensing (QS) mechanism mediated by cell-to-cell signaling molecules, primarily two Acyl-Homoserine Lactones and the Pseudomonas Quinolone System (Bjarnsholt et al., 2010). Once the QS system has been triggered, downstream effector molecules initiate the production of various extracellular molecules including extracellular DNA (eDNA), proteins, polysaccharides, siderophores, and phenazines (pyocyanin) (Bjarnsholt et al., 2010; Flemming and Wingender, 2010; Das et al., 2013b). These extracellular molecules serve multiple functions: they allow establishment of the biofilm matrix, in which bacteria are embedded and protected from physical and chemical challenges, and also act as virulence factors that inhibit/prevent a successful host immune response (Govan and Deretic, 1996; Flemming and Wingender, 2010; Das et al., 2013b).

*Pseudomonas aeruginosa* eDNA is an important extracellular molecule that initiates bacterial adhesion to biotic and abiotic surfaces (Das et al., 2013b). Current research demonstrates that eDNA facilitates biofilm formation by both Gram-negative and Gram-positive bacteria with eDNA acting as an essential factor for initial bacterial adhesion, aggregation, colony formation and for structural integration of the biofilm (Whitchurch et al., 2002; Petersen et al., 2005; Swartjes et al., 2012; Das et al., 2013b). In *P. aeruginosa*, DNA release is mediated by different factors, including phage, *N*-oxo-2-heptyl-4-Hydroxyquinoline (HQNO) and phenazine. These factors induce bacterial cell lysis and trigger release of cytoplasmic DNA into the extracellular environment (eDNA) (Allesen-Holm et al., 2005; Das and Manefield, 2012; Hazan et al., 2016; Turnbull et al., 2016); eDNA may be subsequently released via active membrane vesicles (Kadurugamuwa and Beveridge, 1995). eDNA can bind directly with antibiotics thereby protecting *P. aeruginosa* biofilms by reducing antibiotic penetration (Mulcahy et al., 2008; Chiang et al., 2013; Hazan et al., 2016) and through stimulating antibiotic resistance gene expression (Wilton et al., 2015). Treatment of *P. aeruginosa* biofilms with DNase I (an enzyme that cleaves DNA), significantly disrupts biofilms and enhances antibiotic efficacy (Tetz et al., 2009).

The QS system in *P. aeruginosa* also initiates production of different types of phenazine molecules through activation of the phenazine locus *phzA-G* (Mavrodi et al., 2001). *phzA-G* produces phenazine-1-carboxylic acid (PCA), which is converted to pyocyanin, encoded by *phzM/S* (Mavrodi et al., 2001). PCA also forms others types of phenazines including phenazine-1-carboxamide (encoded by *phzH*) and 1-hydroxyphenazine (*phzS*) (Mavrodi et al., 2001). Data from studies with CF and bronchitis patients infected with *P. aeruginosa*, has demonstrated pyocyanin concentrations up to 85 and 130 μM in their sputum, respectively (Wilson et al., 1988). Pyocyanin also been recorded in significant amount (up to 5.3 μg/g) in wound exudates of burn injury patients infected with *P. aeruginosa* (Muller et al., 2009). Whereas, some recent studies suggest that pyocyanin production level varies considerably among different *P. aeruginosa* isolates (Araújo Jácome et al., 2012; García-Contreras et al., 2015) and this is likely due to host adaptation leading to reduced expression of virulence factors. Pyocyanin is a small heterocyclic compound with biological activities that aid in the development of *P. aeruginosa* biofilm (Price-Whelan et al., 2006). Pyocyanin is a major virulence factor responsible for oxidative stress to lung epithelial cells and ultimately leads to lung damage, respiratory failure and death (O’Malley et al., 2003, 2004). Previous pyocyanin research focused on investigating its virulence in human bronchial epithelial (HBE) cells, the alveolar epithelial A549 cell line, and the CFBE41o-cell line from a CF patient, and *in vivo* in the CD-1 adult mouse model. However, studies have demonstrated that in immune-compromised CF patients pyocyanin induces reactive oxygen species (ROS) production that depletes intracellular glutathione (GSH) levels, leading to widespread epithelial cell death and damage, and persistent biofilm infections (O’Malley et al., 2003, 2004; Lau et al., 2004; Schwarzer et al., 2008).

In this study we ascertained the coordinate role of pyocyanin and eDNA in facilitating biofilm formation by *P. aeruginosa* CF isolates, while establishing the effect of exogenous GSH, DNase I, or antibiotics, on these biofilms and the underlying epithelial cells *in vitro*.

## Materials and Methods

### *P. aeruginosa* Strains Used in This Study

Laboratory strains: PA14 wild-type (Das and Manefield, 2012), the phenazine deficient mutant PA14Δ*phzA-G* (also known as Δ*phz1*/*2* because both pyocyanin gene clusters *phzA-G1* and *phzA-G2* have been deleted), the pyocyanin over-producing strain DKN370 (Dietrich et al., 2008; Ramos et al., 2010) and PAO1 wild-type (ATCC-15692, Das and Manefield, 2012). CF clinical isolates: the Australian Epidemic Strain isolates AES-1R, AES-1M and an adult CF isolate of AES-2 (Manos et al., 2009; Naughton et al., 2011), the Liverpool Epidemic Strains: LESB58 and LES431 (Salunkhe et al., 2005). The strains used, their source and antibiotic profile, are listed in **Table 1**. Note, AES-1M is a chronic infection isolate that is the isotype of the acute infection isolate AES-1R, the latter was isolated from a 14-month-old infant and AES-1M from the same child at 12 years of age. During this time, the strain was not eradicated, and had adapted to the host environment by down regulating virulence factors, including pyocyanin, that stimulate a host immune response (Naughton et al., 2011).

**Table 1:** Source and antibiotic susceptibility of *Pseudomonas aeruginosa* strains used in this study.

*P. aeruginosa strains*	Source (Ref.)	Antibiotic profile					
		
		CIP	TZP	MER	FEP	AMK	GEN
**Cystic fibrosis isolates**							
AES-1R	Naughton et al., 2011	S	I	R	I	R	R
AES-1M		S	I	R	I	R	R
AES-2	Manos et al., 2009	I	R	I	I	R	R
LESB58	Salunkhe et al., 2005	I	S	S	R	R	R
LES431		I	R	R	R	I	I
**Laboratory strains**							
PA14 WT	Das and Manefield, 2012	S	S	S	S	S	S
PA14Δ*phzA-G*	Dietrich et al., 2008; Ramos et al., 2010	NA	NA	NA	NA	NA	NA
DKN370		NA	NA	NA	NA	NA	NA
PAO1 WT	Das and Manefield, 2012	S	S	S	S	S	S


*The sensitive and resistant designations for strains were obtained from standard disk diffusion tables as provided by the Clinical and Laboratory Standards Institute, United States (CLSI-Vol 32, No. 1, 2012). The disk diffusion assay for ciprofloxacin was conducted using a standard disk diffusion assay (Clinical and Laboratory Standards Institute, United States, 2012). CIP, ciprofloxacin; TZP, tazobactam/piperacillin; MER, meropenem; FEP, cefepime; AMK, amikacin; GEN, gentamicin; S, sensitive; I, intermediately sensitive; R, resistant; NA, not available.*

### General Chemicals Used for This Study

Unless indicated otherwise all reagents were obtained from Sigma–Aldrich (Sydney, Australia). Pyocyanin was obtained from Cayman chemicals (Sapphire Biosciences, Sydney, Australia) and DNase I was from Invitrogen (Melbourne, Australia).

### Pyocyanin Production by *P. aeruginosa* Isolates

To quantify pyocyanin production in planktonic culture, single isolated colonies of *P. aeruginosa* strains were inoculated into 5 mL of LB in six-well plates (Corning, Sigma–Aldrich, United States), and incubated at 37°C with shaking at 150 rpm. After 48 h, the bacterial culture was removed, centrifuged (5000 rpm, 5 min at 10°C), and filtered with a 0.22 μm filter (Millipore, United States) to obtain a cell-free supernatant. The cell-free supernatant was used to quantify pyocyanin by absorbance at 691 nm (Das and Manefield, 2012). A standard curve for pyocyanin was generated by addition of a known concentration of purified pyocyanin to LB medium in a 1 mL cuvette and monitoring absorbance at 691 nm (Smartspec 3000 spectrophotometer, Bio-Rad Laboratories, Australia).

### eDNA Quantification in Supernatant of *P. aeruginosa* CF Isolates

eDNA quantification in bacterial cell-free supernatant was analyzed as detailed elsewhere (Das and Manefield, 2012). In brief, cell-free supernatants of *P. aeruginosa* CF isolates AES-1R, AES-1M, AES-2, LESB58, and LES431 were obtained as above and grown for 48 h. The concentration of eDNA present in the filtered supernatant of the isolates was quantified by mixing 20 μL of supernatant with a double stranded DNA quantifying fluorescent dye assay (dsDNABR; Qubit, Invitrogen), and monitored with a Qubit 2.0 Fluorometer (Invitrogen, Life Technologies, Camarillo, CA, United States). Quantification was conducted against a standard curve, yielding an eDNA concentration in μg/mL.

### *P. aeruginosa* Biofilm Biomass Quantification Using Crystal Violet Staining

*Pseudomonas aeruginosa* laboratory strains (PA14 wild-type, Δ*phzA-G*, and DKN370) and CF isolates (AES-1R, AES-1M, and AES-2) were grown in LB medium (24 h, 37°C, 150 rpm) and harvested by centrifugation (5000 × *g*, 5 min at 10°C). After centrifugation, supernatant was removed and the bacterial pellet was suspended in 1 × PBS, 250 μL (OD_600_ = 0.5 ± 0.05) of bacterial cell suspension were added into the wells of 96-well plates (Corning Corp., United States) and incubated at 37°C for 2 h at 150 rpm. After 2 h, wells were gently washed one time with 1 × PBS (to remove any loosely adhered bacteria) from the well surface. To the remaining adhered bacteria 250 μL of LB was added (to initiate biofilm growth) and incubated at 37°C for 24 h at 150 rpm. Where indicated, biofilms were grown in the presence of different modules: pyocyanin (200 μM), DNase I (40 U), DNA (1 ng/μL) or GSH (5 mM), these modules were added to the wells during the final 24 h incubation stage (i.e., biofilm initiation stage). After 24 h of biofilm growth, the LB was gently removed by aspiration and the wells washed two times with 1 × PBS. The biofilm biomass attached to the wells was stained with 0.1 % (w/v) crystal violet (125 μL) and incubated further at room temperature for 60 min followed by washing three times with 1 × PBS to remove excess crystal violet stain. The pre-stained biofilm was dissolved using 30% (v/v) acetic acid in water and transferred into new 96-well plate for biomass quantification at absorbance 550 nm using a Tecan plate reader (Infinite M1000 pro, Sydney Australia).

### *P. aeruginosa* CF Biofilms Viability

Biofilms of *P. aeruginosa* CF isolates (AES-1R, AES-2, LESB58, and LES431) were grown in 96-well plates as described above. After 48 h, biofilm were washed once with 1 × PBS followed by treatment (for 24 h, 37°C, 150 rpm) with different modules: either with ciprofloxacin (5, 10, and 15 μg/mL), DNase I (40 U) or GSH (5 mM) individually or combination: DNase I + Cip (5 μg/mL), GSH + DNase I, GSH + Cip (5 μg/ml), and GSH + DNase I + Cip (5 μg/mL). After 24 h, treated biofilms supernatant were replaced with 200 μL of 1 × PBS, followed by addition of 15 μL resazurin 0.05% w/v solution (Sigma–Aldrich), and incubated further at 37°C, 150 rpm. After 24 h, the fluorescent intensity of the biofilm was determined at Ex_544 nm_ and Em_590 nm_ (Tecan infinite M1000 pro). Notably, resazurin assay works by recording fluorescence intensity (FI) and depends upon two factors (total number of bacteria and total number of viable bacteria in a given biofilm sample).

### Artificial Sputum Media (ASMDM) Preparation

For bacterial growth and biofilm formation study in ASMDM, the media was prepared as previously described (Fung et al., 2010) with the addition of the following: tetracycline (16 μg/mL), penicillin (1 μg/mL), and ampicillin (1 μg/mL). ASMDM media was immediately stored as 10 mL aliquots in sterile 30 mL screw-capped clear glass bottles (McCartney bottles, Sydney Australia) at 4°C until required.

### Confocal Laser Scanning Microscopy of *P. aeruginosa* AES-1R Biofilms Grown in ASMDM

*Pseudomonas aeruginosa* CF isolate AES-1R was grown in LB for 24 h and harvested as described above. Harvested bacteria were re-suspended in 1 × PBS and for initiating biofilm growth in ASMDM, 100 μL (OD_600_ = 0.5 ± 0.05) of bacterial cell suspension were added to 3 mL of ASMDM in a glass Petri dish and incubated with lids on at 37°C with gentle shaking at 150 rpm. After 48 h, biofilms were incubated further at 37°C with gentle shaking at 150 rpm with one of the following treatments: GSH (5 mM), DNase I (40 U), ciprofloxacin (5 μg/mL) individually or a combination: GSH + DNase I, GSH + ciprofloxacin, DNase I + ciprofloxacin or combination of all three: GSH + DNase I + ciprofloxacin. After 24 h incubation, the biofilm growth medium was discarded and the biofilms were washed three times with 1 × PBS to remove any planktonic/loosely bound bacterial cells. Biofilms were then stained with a live/dead stain (Bacterial Viability Kit, Life Technologies) for 30 min in the dark and cells visualized by CSLM (Olympus FV1200, Australia) with Excitation: 473 and 559 nm and Emission: 500 and 637 nm, for syto-9 (green-live) and propidium iodide (red-dead) staining, respectively. ImageJ software was employed to generate images and quantify biovolume, percentage surface area coverage of biofilm and percentage of live and dead biofilm biovolume.

### A549 Lung Epithelial Cell Culture

To determine the effect of pyocyanin, GSSG and GSH on human lung epithelial cells, the A549 cell line (Cellbank Australia, Westmead, Sydney, Australia) (passage #4–12) were cultured in DMEM, supplemented with 10% (v/v) FBS, penicillin (100 IU/mL), and streptomycin (100 μg/mL). A549 cells were maintained in a T-25 cell culture flask (Corning, United States) at 37°C in a 5% (v/v) CO_2_ atmosphere and harvested at 90% confluence using 0.12% v/v trypsin-EDTA. Cells were collected by first quenching Trypsin 1:1 v/v with supplemented media and transferred to conical centrifuge tubes, followed by centrifugation (5 min, 200 × *g*, 20°C). The supernatant was aspirated and the cell pellet was suspended in supplemented DMEM media for further experiments. In some experiments A549 cells were subjected to different treatments classified as follows: (i) Group 1: control (no treatment), 130 μM pyocyanin + 130 μM NADPH, 130 μM pyocyanin + 130 μM NADPH + 2600 μM GSSG, or 130 μM pyocyanin + 130 μM NADPH + 2600 μM GSH and 2600 μM GSSG or GSH alone; (ii) Group 2: control (no treatment), 130 μM pyocyanin + 130 μM NADPH, 130 μM pyocyanin + 130 μM NADPH + 2600 μM GSH and finally cells treated with 2600 μM GSH alone.

### Effect of *P. aeruginosa* Growth in A549 Cells

To study the effect of *P. aeruginosa* CF isolates (AES-1R, AES-2, LESB58, and LES431) on A549 growth and confluence, A549 cells were cultured and harvested as above. After harvesting, cells were plated to a density 6 × 10^5^ cells/mL into six-well plates (Corning) and allowed to incubate for 24 h at 37°C in a 5% (v/v) CO_2_ atmosphere to a confluence of 50%, after which 100 μL of *P. aeruginosa* (OD_600 nm_ = 0.1) suspended in 1 × PBS was introduced into the A549 media and the flask allowed to incubate for a further 48 h. Where indicated, A549 media were also incubated with a combination treatment: (5 mM GSH + 40 U DNase I + 5 μg/mL ciprofloxacin) in the absence and presence of added bacteria. After 48 h the A549 cells in the well plates were imaged using phase contrast microscopy (Zeiss, Axio, Germany) for growth appearance, adherence and confluence.

### Effect of Pyocyanin, GSSG and GSH on Intracellular Total GSH and GSSG

To study the effect of pyocyanin, GSSG and GSH on intracellular levels of total GSH and GSSG, A549 cells were cultured and harvested as above and plated at a cell density of 6 × 10^5^ cells/mL into six-well plates (Corning), and sub-cultured to ca. 90% confluence at 37°C in a 5% (v/v) CO_2_ atmosphere. After 72 h, cells were exposed to group 1 treatments, and sub-cultured at 37°C in a 5% (v/v) CO_2_ atmosphere. After a further 24 h, the total intracellular GSH and GSSG were quantified using a glutathione assay kit manufacturer’s instructions (Cayman chemicals, United States). Briefly, after incubation adherent A549 cells from the six-well plates were harvested by mechanical scraping and centrifuged (200 × *g*, 10 min) to generate a pellet. Cell pellets were subsequently sonicated in 2 mL of cold 50 mM 2-(*N*-morpholinoethanesulfonic acid buffer (MES buffer, pH 7.4) followed by centrifugation (10,000 × *g*, 15 min) at 4°C. The supernatant was then deproteinated using a buffer solution containing metaphosphoric acid (10% w/v) and triethanolamine (53.1% v/v) to remove proteins and avoid interference from protein-sulfhydryl groups. After deproteination the supernatants (sample) were divided into two portions, one was used to determine total GSH and the other assigned to GSSG quantification. For GSSG quantification the sample were further treated with 2-vinylpyridine as per manufacture instruction to derivatizing GSH from the sample. GSH/GSSG quantification: 50 μL of respective samples and 50 μL of standard (Cayman, Sapphire Biosciences, Sydney, Australia) were added to separate wells (*n* = 4 for each sample and standard) in a 96-well plate, followed by addition of 150 μL of freshly prepared GSH assay cocktail (prepared as per the manufacturer’s instructions) to each well containing sample and standard. The plate was covered with the lid and incubated in the dark on an orbital shaker. Total GSH and GSSG absorbance was measured at 5 min intervals for 30 min at A_405_ using an automated plate reader (Tecan infinite M1000 pro). The end point value at 30 min was used to calculate the total intracellular GSH and GSSG concentration in A549 cell lysate.

### Effect of Pyocyanin on A549 Glutathione Peroxidase (GPx) Activity

To study the effect of pyocyanin on cellular glutathione peroxidase (GPx) activity, A549 cells were cultured and harvested and subsequently exposed to the group 1 treatment regimen. After 24 h subculture, total GPx activity was analyzed as described previously (Owens and Belcher, 1965). In brief, 125 μL of master mix (solution A, containing: 1 mM of EDTA, 1 mM Sodium Azide, 1 mM GSH, 0.1 U Glutathione Reductase (GRed), 12.5 mM PBS, 0.25 mM NADPH) was added to wells of a 96-well plate, followed, respectively, by addition of sample (10 μL), and a second Master-mix (65 μL), (solution B, containing: 350 μM H_2_O_2_ prepared in 50 mM 1 × PBS). Spectrophotometric readings were then taken at A_340nm_ (Tecan infinite M1000 pro), immediately after addition of Master-mix B, and 2 min intervals for up to 10 min. The time-dependent change in absorbance was used to calculate the GPx activity of the test samples (Equation 1).

2GSH+H2O2→GPxGSSG+2H2O⁢ GSSG+β−NADPH→G⁢Redβ−NADP++2GSH

### Effect of Pyocyanin on A549 Glutathione Reductase (GRed) Activity

To study the effect of pyocyanin on GRed activity, A549 cells were cultured, harvested and treated as per group 1 above. After 24 h subculture, activity was analyzed using a GRed activity assay kit protocol (Cayman, Sapphire Biosciences, Sydney, Australia). In brief, 20 μL A549 cell lysate (sample) prepared as above, was added to 96-well plates containing 100 μL assay buffer (Cayman, Sapphire Biosciences) and 20 μL GSSG, followed by addition of 50 μL NADPH to initiate the reaction. The plates were immediately shaken for few seconds and a decrease in absorbance at 340 nm was recorded every minute for 10 min, to confirm the oxidation of NADPH to NADP^+^, which is directly related to GRed activity (Equation 1).

### Effect of Pyocyanin, GSSG and GSH on Growth and Confluence of Non-established A549 Cells

Confluent A549 cells were harvested and inoculated (final seed density 10 × 10^5^ cells/mL) into six-well plates, subjected to group 1 treatment, and then sub-cultured for up to 168 h at 37°C and 5% (v/v) CO_2_ during which the effect of pyocyanin, GSSG, and GSH on growth and confluence was monitored and imaged using phase contrast microscopy at 0, 24, 72, 120, and 168 h post-treatment.

### A549 Cell Count

Confluent A549 cells were harvested, and plated at final seed density 10 × 10^5^ cells/mL into 24-well plates (Corning, Sydney Australia) and subjected to group 1 treatment. The cells were then outgrown for up to 168 h at 37°C in a 5% (v/v) CO_2_ atmosphere. The effect of pyocyanin, GSSG, and GSH on A549 cell growth was determined by measuring cell number using EVE^TM^ Automated Cell Counter, NanoEnTek and Cell Counting Slides at the beginning (0 h) and at all other time points (24, 72, 120, and 168 h).

### Effect of Pyocyanin and GSH on A549 Viability

The effect of pyocyanin and GSH on A549 cell viability was determined using a Resazurin assay. Confluent A549 cells were harvested, were plated at final seed density 10 × 10^5^ cells/mL into 24-well plates (Corning, Sydney, Australia). Separate plates were then incubated at 37°C in a 5% (v/v) CO_2_ and harvested at 24, 72, 120, and 168 h post-treatment, while another plate was treated and assessed immediately for the zero time point. At each time point, the cells were removed from incubation, treated with 10 μL of a 0.05% w/v Resazurin solution (Sigma–Aldrich), and incubated further at 37°C with gentle shaking (150 rpm). After 24 h, the FI of the A549 cells was determined at Ex_544nm_ and Em_590nm_ using the Tecan infinite M1000 pro plate reader.

### Real-Time Monitoring of A549 Confluence

To monitor the effect of pyocyanin and GSH on confluence of A549 cells in real time, the harvested cells (seed density 10 × 10^5^ cells/mL) were added to 96-well plates (Corning, Sydney, Australia) and immediately exposed to group 1 treatments. The 96-well plates were then placed into the IncuCyte Zoom live cell imaging system (Essen BioScience, United States) at 37°C and 5% (v/v) CO_2_ for 240 h. Images of the A549 cells were captured at start (0 h) and then at 24 h intervals until the end of the experiment (240 h), using the standard inbuilt imaging system. The % of cell confluence at different time points was calculated using the inbuilt software.

### Effect of Pyocyanin and GSH on A549 Cell Eccentricity

Harvested A549 cells (seed density 10 × 10^5^ cells/mL) were added to 96-well plates and immediately exposed to group 1 treatments. The 96-well plates were then placed into the IncuCyte Zoom live cell imaging system at 37°C and 5% (v/v) CO_2_ for up to 120 h. Images of the A549 cells were captured at 0 h and then at every 24 h for up to 120 h using the inbuilt imaging system and the A549 cell eccentricity was also calculated using an inbuilt software.

### Effect of Pyocyanin and GSH on Growth and Confluence of Pre-established A549 Cells

Harvested A549 cells were grown to 90% confluence on six-well plates as detailed above. Next, cells were subjected to group 2 treatments and re-incubated for a further 24, 72, and 120 h at 37°C. Cells treated with 130 μM pyocyanin + 130 μM NADPH was also subjected to 2600 μM GSH every 24 h for 72 and 120 h, respectively. Daily GSH treatment was carried out by adding GSH (once every 24 h) directly into the existing media overlying the confluent cells and pyocyanin. No media was removed or replaced and the volume added had negligible impact on the concentration of constituents. The effect of pyocyanin and GSH on cell growth and confluence was monitored and imaged using phase contrast microscopy; OD_570 nm_ of A549 cells were also determined in parallel using a plate reader (Tecan infinite M1000 pro) at 0, 24, 72, and 120 h.

### Detection of Oxidative Stress in A549 Cells as an Effect of Pyocyanin and GSH Treatment

Harvested A549 cells were grown as above to 90% confluence on six-well plates for 72 h as detailed above. After 72 h the cells were subjected to different treatments: control (no treatment), 130 μM pyocyanin + 130 μM NADPH, 130 μM pyocyanin + 130 μM NADPH + 2600 μM GSH, or 2600 μM GSH alone, and re-incubated for further 72 and 120 h at 37°C. Where indicated, cells treated with 130 μM pyocyanin + 130 μM NADPH were also subjected to 2600 μM GSH every 24 h for 72 and 120 h. After 72 and 120 h treatments, levels of oxidative stress was determined using CellROX deep red reagent (Invitrogen, United States), for all treated and untreated (control) cells. Briefly, CellROX reagent at a final concentration of 5 μM was added directly to A549 cells and incubated for 45 min in the dark, before image capture at Ex_644nm_ and Em_665nm_ using a Cytation 3 cell imaging multi-mode reader (BioTek, United States). CellROX deep red reagent reacts with ROS to yield bright red fluorescence.

To quantify FI (i.e., the surrogate measure for oxidative stress) in A549 cells exposed to different treatment conditions, cells were harvested confluent and exposed to pyocyanin and GSH as detailed above, in 24-well plates. Daily treatments with 2600 μM GSH were then performed for 5 days (120 h). After 120 h, the level of FI in the A549 cells under different treatment conditions was determined using CellROX deep red reagent as per manufacture instruction using a plate reader (Tecan infinite M1000) at Ex_644 nm_ and Em_665 nm_. An increase in fluorescence value directly relates to increased oxidative stress in A549 cells.

## Results

### Pyocyanin Expression in *P. aeruginosa* CF and Laboratory Reference Strains

**FIGURE 1:**
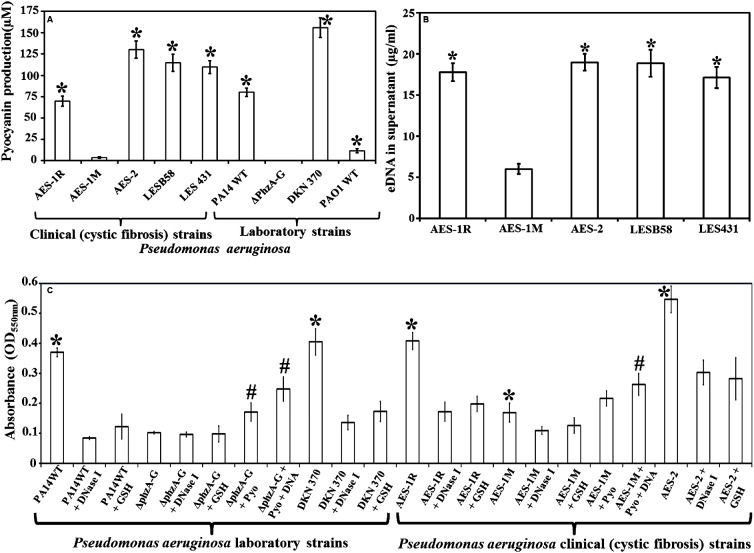
Pyocyanin and eDNA production and biofilm formation by clinical and laboratory strains of *Pseudomonas aeruginosa*. **(A)** Pyocyanin production by *P. aeruginosa* cystic fibrosis isolates: the isogens AES-1R and AES-1M (acute and chronic, respectively); AES-2, LESB58 and LES431 and laboratory strains (PA14 WT, PA14Δ*phzA-G*, DKN370, PAO1WT). Among clinical isolates AES-1M expressed the least pyocyanin (ca. 5 μM), whereas the AES-2 isolate expressed the most (ca. 130 μM). Amongst laboratory strains, the phenazine deficient mutant (PA14Δ*phzA-G*) did not express pyocyanin while the pyocyanin overproducing strain (DKN 370) recorded ∼155 μM pyocyanin. Pyocyanin concentration was measured using standard pyocyanin concentration (μM) vs. 691 nm (OD_691 nm_). **(B)** eDNA production, quantified *P. aeruginosa* supernatants from cystic fibrosis (CF) isolates and pyocyanin producing strains showed a significant increase in eDNA production (17–19 μg/mL) in comparison to AES-1M (6 μg/mL). **(C)** Crystal violet assay showing biofilm biomass of *P. aeruginosa* laboratory and clinical strains grown in presence or absence of glutathione (GSH), DNase I, exogenous pyocyanin and exogenous DNA to complement DNase I activity. Strains deficient of pyocyanin production formed significantly smaller biofilm biomasses, whereas addition of pyocyanin (200 μM) and DNA (1 ng/μL) facilitated a significant increase in biofilm biomass. DNase I or GSH-treatment of PA14, pyocyanin over-producing strain DKN 370 and the clinical isolates AES-1R and AES-2 resulted in biofilms similar in size to those of PA14Δ*phzA-G* and AES-1M. **(A)** ^∗^*P* < 0.05 when compared to AES-1M and Δ*phzA-G*, **(B)** ^∗^*P* < 0.05 when compared to AES-1M, and **(C)** ^∗^*P* < 0.05 when compared to its GSH and DNase I treated counterpart. ^#^*P* < 0.05 when compared to untreated PA14Δ*phzA-G* and AES-1M. Data represent mean ± SD; *n* = 3 experiments performed in biological replicate.

Pyocyanin production varied amongst the different strains *of P. aeruginosa* tested by growth in LB for 48 h (**Figure 1A**). Among CF isolates, AES-1M produced the lowest amount of pyocyanin (∼5 μM) followed by AES-1R (∼60 μM) whereas AES-2 and LES strains produced more than 100 μM of pyocyanin. Among laboratory strains, the pyocyanin deficient mutant Δ*phzA-G* did not produce pyocyanin whereas as anticipated the pyocyanin over-producing mutant DKN 370 produced the most pyocyanin (>150 μM). Strains PA14WT and PAO1WT produced about 70 and 10 μM pyocyanin, respectively.

### eDNA Production in *P. aeruginosa* CF Isolates Cell-Free Supernatant

eDNA production in *P. aeruginosa* is dependent upon pyocyanin expression. **Figure 1B** shows high pyocyanin producing CF isolates AES-1R, AES-2, LESB58, LES431 produced significantly (*P* < 0.01) high amounts of eDNA in their supernatant (18, 19, 19, and 17 μg/mL, respectively) whereas the low pyocyanin expressing strain AES-1M produced only 6 μg/mL eDNA.

### Effect of Pyocyanin, GSH and DNase I on the Biofilm Biomass of *P. aeruginosa* Strains

**Figure 1C** shows the influence of DNA, pyocyanin and GSH on biofilm formation of the various *P. aeruginosa* laboratory and clinical strains grown in LB for 24 h. Biofilm formation was analyzed by measuring the total biofilm biomass using a crystal violet assay. PA14 grown in the presence of DNase I or GSH alone had a significantly lower biofilm biomass (*P* < 0.05; OD_550 nm_ = 0.09 and 0.13, respectively) compared to untreated PA14 (OD_550 nm_ = 0.37). DKN370 also showed a significant (*P* < 0.05) reduction in biofilm biomass when grown in the presence of DNase I or GSH. On the other hand, the phenazine deficient mutant (PA14Δ*phzA-G*) formed a biofilm biomass similar in size (OD_550 nm_ = 0.096) to DNase I or GSH-treated PA14 biofilms. However, when PA14Δ*phzA-G* was supplemented with pyocyanin (200 μM) or with pyocyanin + exogenous DNA (1 ng/μL) it showed a significant (*p* < 0.05) increase in biofilm biomass (OD_550 nm_ = 0.17 and 0.24, respectively) compared to untreated, DNase I or GSH-treated PA14 Δ*phzA-G*. With respect to CF isolates, the low pyocyanin producing chronic infection isogen AES-1M formed significantly smaller biofilm biomass (OD_550 nm_ = 0.17, *P* < 0.05) than its acute isogenic counterpart AES-1R (OD_550 nm_ = 0.41) and the isolate from strain AES-2 (OD_550 nm_ = 0.55). When AES-1R and AES-2 biofilms were grown in the presence of DNase I, they formed significantly smaller biofilms (DNase I: OD_550 nm_ = 0.17 and 0.30, *P* < 0.05, respectively), (GSH: OD_550 nm_ = 0.19 and 0.28, respectively). DNase I also induced a small but significant decrease in biofilm biomass in AES-1M (*p* > 0.05). Interestingly, addition of exogenous pyocyanin (200 μM) or pyocyanin + 1 ng/μL DNA led to a significant increase in AES-1M biofilm biomass (OD_550nm_ = 0.22 and 0.26, *P* < 0.05, respectively) in comparison to the biomass of AES-1M alone.

### Effect of Combination Therapy on Mature Biofilm*s* of *P. aeruginosa* CF Isolates

**FIGURE 2:**
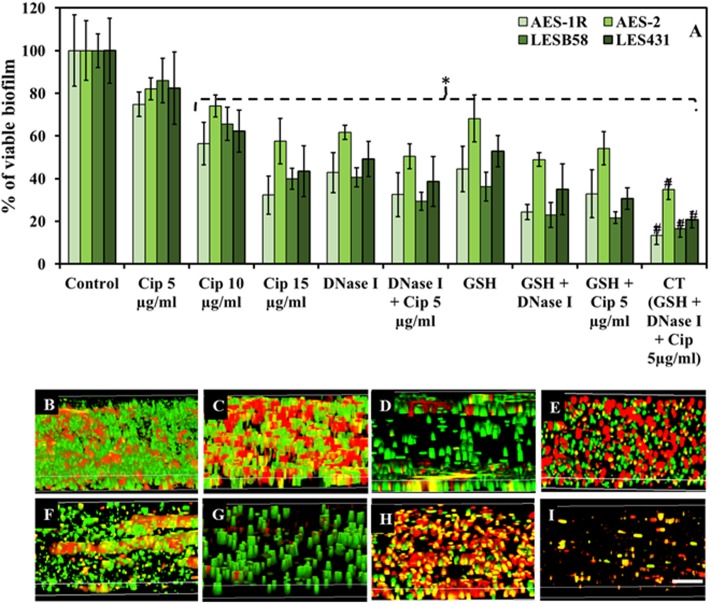
Effect of GSH, DNase I, ciprofloxacin and combination therapy (CT) on *P. aeruginosa* biofilm. **(A)** The resazurin bacterial viability assay showed effect of ciprofloxacin, GSH, DNase I and combination treatment on 48 h old established biofilms of different *P. aeruginosa* CF isolates grown in LB media. All isolates showed significant decrease in bacterial viability when treated with ciprofloxacin (Cip) at the highest concentrations (10 and 15 μg/mL). Conversely, DNase I or GSH-alone treatment and in combination with low Cip concentration (5 μg/mL), resulted in a significant decrease in *P. aeruginosa* viability. The most significant decrease in bacterial viability was observed with biofilms subjected to all three components of the CT GSH + DNase + Cip 5 μg/ml. **(B–I)** Confocal microscopy imaging showing the effect of ciprofloxacin, GSH, DNase I and GSH-combination treatment, on 48 h old established biofilms of AES-1R grown in ASMDM. With untreated AES-1R biofilm **(B)**, ciprofloxacin alone did not disrupt the biofilm but showed an increase in red-staining (dead) cells **(C)**. Disruption of the biofilm architecture of AES-1R was observed when it was treated with DNase I **(D)** or DNase I combined with ciprofloxacin **(E)**. Similarly, GSH alone **(F)** and in combination with either DNase I **(G)**, or ciprofloxacin **(H)**, increased biofilm disruption and dead bacterial cells. Combination therapy (GSH + DNase I + Cip 5 μg/mL) resulted in complete disruption of the biofilm (I). Scale bar = 50 μm. **(A)** ^∗^*P* < 0.05 when compared to control. ^#^*P* < 0.005 when compared to all treatments. Data represent mean ± SD; *n* = 3 experiments performed in biological replicate.

Combination Therapy (CT) (GSH + DNase I + ciprofloxacin 5 μg/mL) showed the highest efficacy in affecting biofilm viability, **Figure 2A**. CF isolates AES-1R, AES-2, LESB58, and LES431 showed biofilm viabilities between 75 and 86, 56 and 74 and 32 and 58% in the presence of increasing doses of ciprofloxacin (5, 10, and 15 μg/mL), respectively, but with only 10 and 15 μg/mL ciprofloxacin reaching statistically significant (*P* < 0.05) decreases in viability compared to control/untreated biofilm. Biofilm viability decreased significantly (*P* < 0.05) when treated with DNase I (43–62%) and GSH (45–68%) individually and GSH + DNase I combined (24–49%). A combination of low (5 μg/ml) ciprofloxacin with DNase I or GSH significantly (*P* < 0.05) decreased biofilm viability (32–51 and 33–54%, respectively). Interestingly, the most significant (*P* < 0.05) decrease in biofilm viability (13–35%) was observed in the presence of CT. Notably, DNase I is non-bactericidal, but a decrease in biofilm fluorescence (i.e., biofilm viability) in the presence of DNase I is mainly due to disruption (removal) of biofilm biomass after treatment. Low biofilm also led to low FI in the resazurin assay, as indicated in “Materials and Methods.”

### CT Modulates Biofilm Architecture of *P. aeruginosa* CF Isolate AES-1R

Biofilm architecture and viability of *P. aeruginosa* AES-1R changed significantly when subjected to different treatments, **Figures 2B–I**. The effect of 5 mM GSH, 40 U DNase I, and 5 μg/mL ciprofloxacin individually or in combination (two or three components) on established biofilms of AES-1R grown on ASMDM is shown in **Figures 2B–I**. Live/dead biofilm imaging showed considerable disruption in biofilm architecture of AES-1R when treated for 24 h with GSH (**Figure 2F**) or DNase I (**Figure 2D**) alone. However, treatment with ciprofloxacin (**Figure 2C**) alone did not disrupt the biofilm, despite the fact that more dead (red) biofilm cells were detected than in the corresponding control. Conversely, combinations of DNase I + ciprofloxacin (**Figure 2E**), GSH + ciprofloxacin (**Figure 2H**), GSH + DNase I (**Figure 2G**) and CT (**Figure 2I**) enhanced biofilm disruption and modulated more changes in biofilm architecture than in the corresponding individual treatment regimens and control.

### The Effect of CT on *P. aeruginosa* CF Isolates Growing on Lung Epithelial Cells A549

**FIGURE 3:**
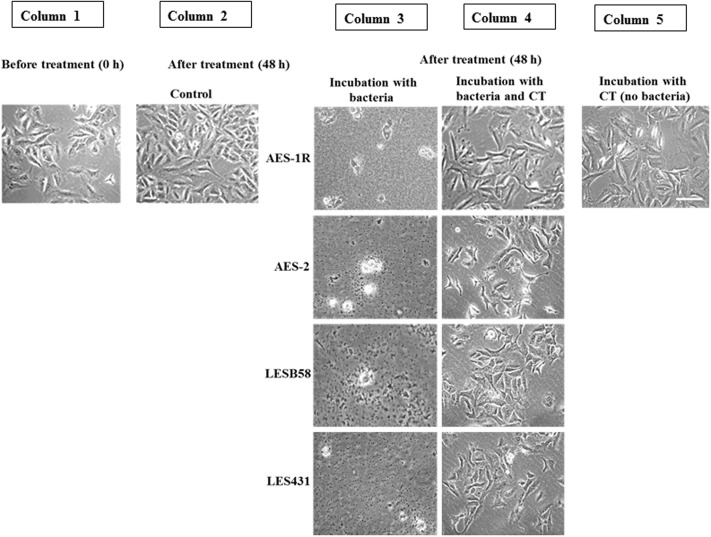
Effect of CT on A549 lung epithelial cells infected with *P. aeruginosa* CF strains. Uninfected confluent 24 h-old A549 cells (Column 1) were infected with the above *P. aeruginosa* strains for 48 h. This resulted in the destruction of the A549 cell architecture and colonization of the remains by the bacteria (Column 3). When, A549 cells were incubated with bacteria and CT together, A549 cells were still adherent and maintained their confluence on well plates, with no evidence of bacterial colonization (Column 4), 48 h incubation with CT alone showed maintenance of almost full A549 confluence, (Column 5) similar to the untreated control (Column 2). Scale bar = 50 μm. Image shown is representative of all results, *n* = 3 experiments performed in biological replicate.

**Figure 3** shows that CT inhibited growth and biofilm formation in CF isolates while facilitating A549 cell growth. A549 cells initially grown for 24 h in DMEM cell culture media (to allow 50% confluence) were subjected to: (i) 48 h incubation of A549 without any treatment (control), showing almost complete coverage of cells on surface, (ii) 48 h incubation with CF isolates AES-1R, AES-2, LESB58, and LES431, resulting in bacterial colonization and A549 cells losing adherence and growth, (iii) 48 h incubation with different CF isolates + CT, resulting in A549 cells remaining adherent (about 60% confluence), and (iv) 48 h incubation with CT alone, resulted in the A549 cells remaining adherent similar to control.

### Effect of Pyocyanin, GSSG and GSH on A549 Confluence and Viability

**FIGURE 4:**
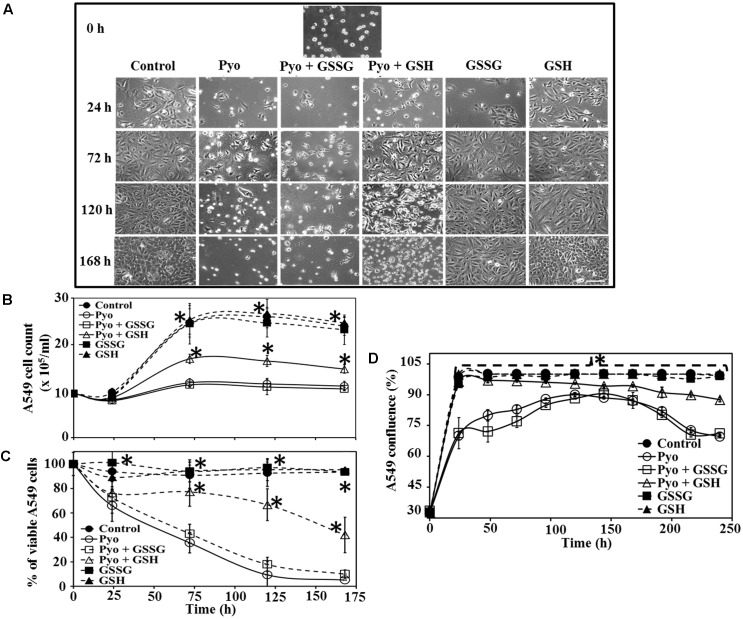
Effect of pyocyanin, GSSG, and GSH on A549 cell growth and confluence over different time periods **(A)**. A549 adherence and confluence in six-well plates at 24, 72, 120, and 168 h post-treatment, with example of adherence at 0 h. Pyocyanin considerably reduced the confluence of A549 and pyocyanin + GSSG did not increase A549 confluence; however, addition of pyocyanin + GSH resulted in an increase in A549 confluence. GSSG or GSH alone resulted in A549 confluence similar to that of the control. Scale bar = 50 μm. **(B)** A549 cells exposed to pyocyanin showed a significant decrease in cell number, to a level similar to that of pyocyanin + GSSG, while pyocyanin + GSH showed a significant increase in cell number, but still lower than the untreated control, GSSG and GSH-alone. **(C)** Resazurin assay showed pyocyanin exposed A549 cells rapidly decreased in viability to a level similar to pyocyanin + GSSG, whereas, A549 cells treated with pyocyanin + GSH showed a significant increase in viability. The percentage of live/respiring cells remained at more than 90% for untreated control, GSSG and GSH alone, from 0 to 168 h. **(D)** The percentage of A549 confluence over the 240 h period showed a significant increase in confluence for the control, GSH and pyocyanin + GSH-cells compared to pyocyanin alone and pyocyanin + GSSG, at all time-points. **(B–D)** ^∗^*P* < 0.05 compared to A549 grown in presence of pyocyanin and pyocyanin + GSSG. Data represent mean ± SD; *n* = 3 experiments performed in biological replicate.

The effect of GSH on A549 adherence and confluence on the surface of six-well plates was examined at different times post-treatment and under different growth conditions. Microscopy showed that in the presence of pyocyanin and pyocyanin + GSSG, the confluence of A549 cells decreased (especially at 72, 120, and 168 h), while addition of pyocyanin + GSH resulted in an increase in A549 confluence. On the other hand, when GSSG or GSH alone were added, A549 confluence was similar to that of the control (**Figure 4A**). **Figure 4B** shows the cell count of A549 cells after addition of pyocyanin, GSSG, and GSH. In the presence of pyocyanin and pyocyanin + GSSG the A549 cell count increased marginally after 168 h (total cell density 11.0 and 10.4 × 10^5^ cells/mL, respectively) in comparison to control 0 h (total cell density 9.5 × 10^5^ cells/mL) time point. However, when A549 cells were treated with pyocyanin + GSH, the cell count increased significantly (*P* < 0.05; 14.7 × 10^5^ cells/mL) after 168 h to a level higher than that for A549 cells grown in the presence of pyocyanin. Addition of GSSG or GSH alone resulted in an A549 cell count similar to that of the untreated A549 control (24.0 × 10^5^ cells/mL) after 168 h.

Data in **Figure 4C** show A549 cell viability assessed using the resazurin assay. Cells treated with pyocyanin or pyocyanin + GSSG showed a significant decrease in % of live/respiring cells with ca. 10% live cells detected by 168 h, however, addition of pyocyanin + GSH showed a significant (*P* < 0.05) increase (42%) in A549 viability. The viability of the untreated (control) cells and cells treated with GSSG or GSH alone remained at >90% live/respiring cells over the period 0–168 h. Quantification of the percentage of A549 confluence over a 10 days (240 h) time period is shown in **Figure 4D**. Here, cells exposed to pyocyanin alone and pyocyanin + GSSG showed a significant (*P* < 0.05) decrease in confluence (<75%) by 240 h relative to the untreated control (cell confluency 100%) and all other treatments: pyocyanin + GSH (∼90%), GSSG (100%), and GSH (100%).

### Role of GSH in Balancing GPx and GRed Activity in A549 Cells

**FIGURE 5:**
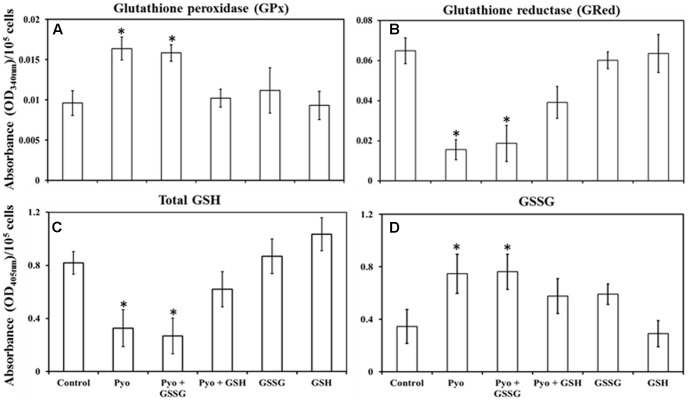
Quantification of glutathione peroxidase, glutathione reductase, total glutathione, and glutathione disulphide in pre-established A549 cells. **(A)** A549 cells treated for 24 h with pyocyanin or pyocyanin + GSSG showed a significantly higher glutathione peroxidase (GPx) activity compared to control, pyocyanin + GSH, GSSG and GSH. **(B)** Glutathione reductase (GRed) activity of pyocyanin and pyocyanin + GSSG treated A549 cells was significantly lower compared to control, pyocyanin + GSH, GSSG and GSH-treated cells. **(C)** Total intracellular GSH levels of A549 cells exposed to pyocyanin and pyocyanin + GSSG were significantly lower compared to A549 cell exposure to control, pyocyanin + GSH or GSSG or GSH. **(D)**. Intracellular GSSG level was elevated in pyocyanin and pyocyanin + GSSG treated A549 cells in comparison to control, pyocyanin + GSH, GSSG or GSH-alone treatment. **(A–C)** ^∗^*P* < 0.05 compared to treatment with control, pyocyanin + GSH, GSSG and GSH. **(D)** ^∗^*P* < 0.05 compared to treatment with control and GSH. Data represent mean ± SD; *n* = 4 biological replicates.

Measurement of intracellular GPx activity showed that pyocyanin and pyocyanin + GSSG treated A549 cells exhibited significantly (*P* < 0.05) higher GPx activity (OD_340 nm_ = 0.016 and 0.015/10^5^ cells/mL, respectively) relative to the control (0.009/10^5^ cells/mL), pyocyanin + GSH (0.012/10^5^ cells/mL), GSSG (0.011/10^5^ cells/mL), and GSH (0.009/10^5^ cells/mL) (**Figure 5A**). By contrast, **Figure 5B** shows GRed activity of A549 cells was significantly (*P* < 0.05) lower in pyocyanin (0.015/10^5^ cells/mL) and pyocyanin + GSSG (0.019/10^5^ cells/mL) treated A549. A549 treated with pyocyanin + GSH showed significantly elevated GRed activity (0.039/10^5^ cells/mL) in comparison to pyocyanin and pyocyanin + GSSG, whereas A549 treated with GSSG (0.060/10^5^ cells/mL) or GSH (0.063/10^5^ cells/mL) alone showed GRed activity levels similar to the control (0.065/10^5^ cells/mL).

### Effect of GSH in Restoring Total Intracellular GSH and GSSG Levels in A549 Cells

Glutathione treatment surpassed the effect of pyocyanin and facilitated an increase in total intracellular GSH and a corresponding decrease in GSSG levels, **Figures 5C,D**. Data in **Figure 5C** showed that the total intracellular GSH level of untreated (control) A549 cells was about 0.8 μM/10^5^ cells/mL which was significantly (*P* < 0.05) higher than intracellular GSH levels in cells exposed to pyocyanin and pyocyanin + GSSG treated A549 (0.3 and 0.3 μM/10^5^ cells/mL, respectively). Interestingly, when A549 cells were exposed to GSH treatment (pyocyanin + GSH), the total intracellular GSH level was restored (0.6 μM/10^5^ cells/mL), while A549 treatment with GSSG or GSH individually showed a small increase (significant at *P* < 0.05 only for GSH treatment) in total intracellular GSH level (0.9 and 1.0 μM/10^5^ cells/mL, respectively) compared to the control. In contrast, intracellular GSSG levels went down significantly (*P* < 0.05) in the control (0.3 μM/10^5^ cells/mL) in comparison to pyocyanin (0.8 μM/10^5^ cells/mL) or pyocyanin + GSSG (0.8 μM/10^5^ cells/mL) and pyocyanin + GSH (0.57 μM/10^5^ cells/mL) treated cells. GSSG-alone treatment also showed a significantly high GSSG level (0.59 μM/10^5^ cells/mL), but for cells exposed to GSH alone, intracellular A549 GSSG levels were only (0.3 μM/10^5^ cells/mL), similar to GSH levels in the control (**Figure 5D**).

### Effect of Pyocyanin and GSH on A549 Cell Eccentricity

**FIGURE 6:**
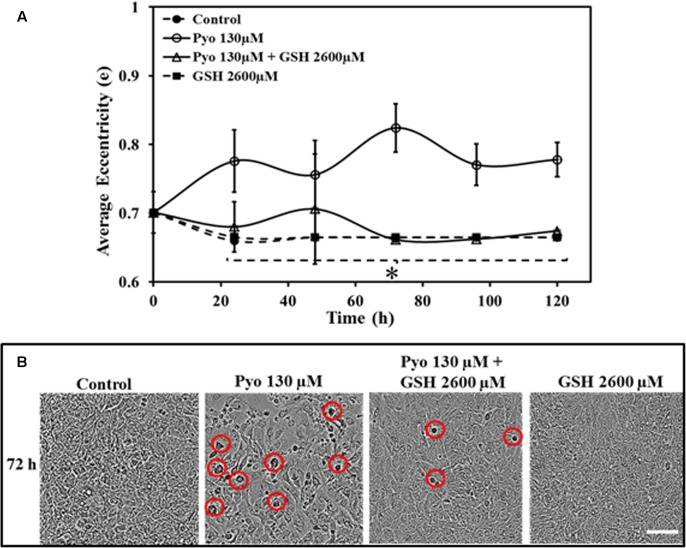
Effect of pyocyanin and GSH on A549 cell eccentricity over different time periods, monitored using IncuCyte Zoom live cell imaging. **(A)** Quantification of the average eccentricity of A549 confluence over 120 h. Control (untreated), GSH alone and pyocyanin + 2600 μM GSH-treated cells showed stable eccentricity, and the difference is significant in comparison to pyocyanin alone at all time-points. **(B)** Example showing 72 h images of A549 adherence, confluence and eccentricity in 96-well plates grown under different conditions. In comparison to the control, GSH alone and the pyocyanin + 2600 μM GSH, the pyocyanin only treated A549 cells showed considerably change in cell morphology (eccentricity-red circle). **(A)** ^∗^*P* < 0.05 when compared to A549 cells grown in presence of pyocyanin. Bar = 50 μm. Results are shown as mean ± SD and all experiments were conducted in biological triplicate (*n* = 3).

A549 cells grown in the presence of pyocyanin displayed significantly different eccentricity when compared to control, GSH alone and pyocyanin + 2600μM GSH-treated cells at all-time points. Control and GSH exposed cells were more stable throughout the study, whereas pyocyanin + GSH-treated cells shows some inconsistency in their eccentricity (**Figure 6A**). **Figure 6B** shows an example of cell morphology at 72 h when grown under different conditions. After 72 h, pyocyanin-only treated A549 cells showed marked alteration of cell morphology (eccentricity-red circle) in comparison to the control, GSH-alone and pyocyanin + 2600 μM GSH treatments.

### Effect of Pyocyanin and GSH on Pre-established A549 Cells

**FIGURE 7:**
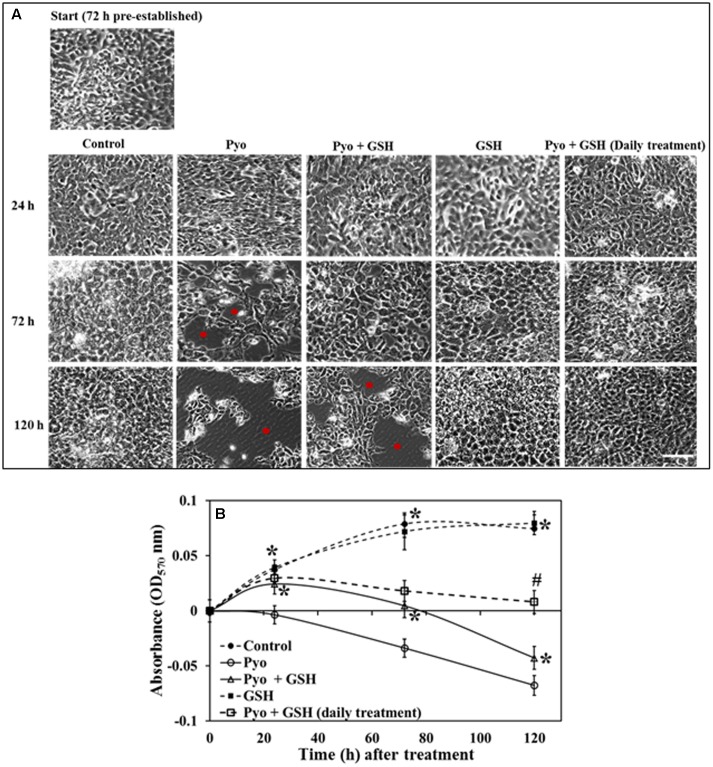
Effect of pyocyanin and GSH on 72 h old pre-established A549 cells. **(A)** Phase contrast microscopy of 72 h pre-established A549 cells subjected to a one-time treatment with pyocyanin resulted in decrease in A549 adherence levels and confluence especially over both the 3 and 5 days periods, whereas treatment with pyocyanin + GSH resulted in decrease in adherence and confluence over 5 days. Red spots in images indicate empty spaces on well plate surfaces due to non-adherence of cells. Treatment of A549 cells with GSH alone resulted in a confluence level similar to that of the untreated control. Interestingly, pyocyanin + GSH at day 1, followed by daily treatment with GSH for 3 and 5 days resulted in increased recovery of A549 adherence and confluence compared to pyocyanin + one-time GSH treatment. Bar = 50 μm. **(B)** OD_570 nm_ of A549 cells quantified from microscopy images confirms that pyocyanin resulted in a significant decrease in A549 cell density over 5 days compared to control, GSH and pyocyanin + GSH at all three time points. Daily GSH treatment resulted in a significant increase in OD in comparison to a pyocyanin + single GSH treatment. **(B)** ^∗^*P* < 0.05 compared to pyocyanin treatment and ^#^*P* < 0.05 compared to pyocyanin + single GSH treatment. Data represent mean ± SD; *n* = 3 independent experiments.

Data show in **Figure 7** indicated that while pyocyanin depleted A549 cell adherence and confluence, GSH treatment stabilized and maintained A549 cell confluence and growth. Pyocyanin, pyocyanin + GSH, and GSH alone, were added to establish 72 h A549 cells. When subjected to pyocyanin or pyocyanin + GSH in a one-time treatment, A549 cells showed a significant decrease in adherence and confluence. This was particularly evident when compared to untreated control cells or cells exposed to GSH alone. Thus, adherence and confluence was altered over the 72 h period (OD_570 nm_ of control = 0.08, pyocyanin = -0.033, pyocyanin + GSH = 0.005, respectively, GSH alone = 0.072) and over 120 h periods (OD_570nm_ of control = 0.075, pyocyanin = -0.07, pyocyanin + GSH = -0.042, GSH alone = 0.08) as shown in **Figures 7A,B**. Interestingly, when we subjected confluent A549 cells (pre-cultured for 72 h) to pyocyanin + GSH at day 1, followed by daily treatment with 2600 μM GSH at 72 and 120 h, a significant (*P* < 0.05) recovery of A549 adherence and confluence (in comparison to pyocyanin + one-time GSH treatment) was identified (**Figure 7A**); OD_570 nm_ measurements (**Figure 7B**) were 0.02 and 0.009, after daily GSH treatment for 72 and 120 h, respectively.

### Oxidative Stress Levels in Pyocyanin and GSH-Treated A549 Cells

**FIGURE 8:**
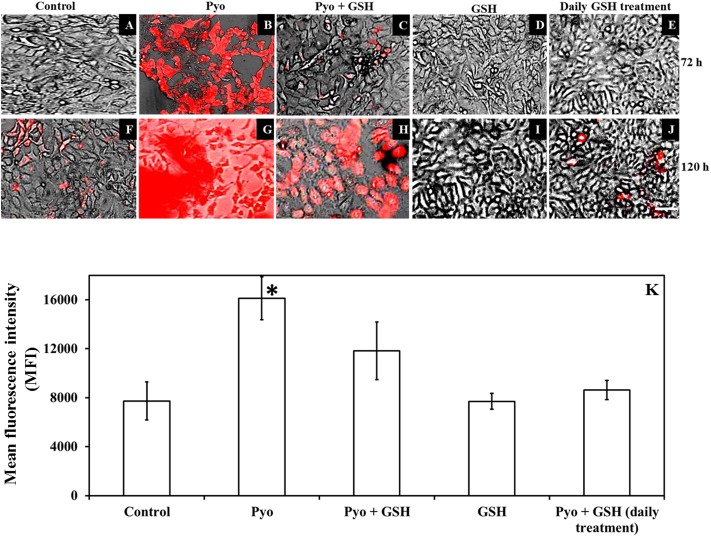
Effect of pyocyanin and GSH on a marker of oxidative stress within A549 cells. The degree of oxidative stress in 72 h old pre-established A549 cells subjected to pyocyanin, pyocyanin + GSH or GSH over 3 and 5 days was examined using the CellROX oxidative stress assay. Extent of red color indicates level of oxidative stress. **(B,G)**: 72 h pre-established A549 were considerably stressed when incubated with pyocyanin for 3 and 5 days in comparison to control/untreated A549 cells **(A,F)**. **(C,H)**: Pyocyanin + GSH resulted in a considerable reduction in oxidative stress. **(D,I)**: GSH did not result in oxidative stress even after 5 days. **(E,J)**: When A549 cells were treated with pyocyanin + GSH followed by treatment with GSH every 24 h for 3 (72 h) and 5 days (120 h), a dramatic reduction in oxidative stress was noted compared to a single treatment with pyocyanin + GSH. Bar = 50 μm. Images shown are representative of results taken from *n* = 3 independent experiments. **(K)** Shows mean fluorescence intensity (MFI) of A549 cells after they were subjected to different treatment regimens. Pyocyanin-alone treated cells exhibited significantly higher (MFI = 16115) relative to control (MFI = 7732) and pyocyanin + single-GSH treatment (MFI = 11833) and GSH alone treatment (MFI = 7700). Whereas, pyocyanin treated A549 cells subjected to daily GSH treatment showed MFI = 8619 slightly higher (insignificant) than control **(K)**. ^∗^*P* < 0.05 compared to all other treatments. Data represent mean ± SD; *n* = 4 independent experiments.

**Figure 8** shows that GSH inhibited pyocyanin mediated oxidative stress in A549 cells as determined by the CellROX assay. Images (**Figures 8A–J**) shows pre-established, confluent, A549 cells subjected to pyocyanin exhibited higher levels of red fluorescence than cells exposed to pyocyanin + GSH treatments at 72 and 120 h post-treatment. Interestingly, pyocyanin treated A549 cells subjected to daily treatment with 2600 μM GSH as described earlier in **Figure 7B**, showed a marked decrease in oxidative stress, reaching levels similar to the untreated A549 control. The quantification of FI of A549 cells (**Figure 8K**) indicated that cells treated with pyocyanin alone exhibited significantly higher oxidative stress (with mean fluorescent intensity; MFI = 16115) relative to background levels detected in control (MFI = 7732), pyocyanin + one-time GSH treated (MFI = 11833) and GSH alone treatment (MFI = 7700). Whereas, pyocyanin treated A549 cells subjected to daily treatment with 2600 μM GSH showed insignificant increase in MFI (8619) relative to control.

## Discussion

Many of the virulence-related roles of pyocyanin have been well studied (O’Malley et al., 2003, 2004; Lau et al., 2004; Schwarzer et al., 2008), however; recent evidence has demonstrated that it is also an important biofilm promoting factor (Das et al., 2015). It has also been reported that phenazine molecules do not affect *P. aeruginosa* adhesion to surfaces, but they significantly lower bacterial swarming motility and facilitate development of structured biofilms with both increased biomass and larger colony size (Ramos et al., 2010). In this study, we showed for the first time that eDNA production in *P. aeruginosa* CF isolates is directly dependent on pyocyanin expression (**Figures 1A,B**) and thereby biofilm formation (**Figure 1C**). Pyocyanin induces eDNA production through H_2_O_2_, as recorded previously in *P. aeruginosa* PA14 (Das and Manefield, 2012) and binds with eDNA via intercalation and presence of eDNA on the bacterial cell surface, whereupon it is able to alter bacterial cell hydrophobicity and the physico-chemical forces that drive bacterial interactions (Das et al., 2013a, 2015). Thus presence of both pyocyanin and eDNA enhances *P. aeruginosa* biofilm formation, as shown in **Figure 1C**.

Our results (**Figure 2**) firstly confirmed that well-established biofilms of CF isolates grown in LB and ASMDM were significantly disrupted and architecturally altered by GSH and that this activity could be due to inhibition of pyocyanin binding to eDNA by GSH (Das et al., 2015). Inhibition of pyocyanin-eDNA binding by GSH can significantly hinder biofilm development, and this likely occurs by its modulation of the physico-chemical interactions central to pyocyanin’s biofilm forming activity. On the other hand, DNase I-mediated biofilm disruption occurs through cleaving of eDNA in biofilms (Whitchurch et al., 2002; Swartjes et al., 2012) while the antibiotic alone had no disruptive effect on the biofilm. Notably, a combination of two of the components (GSH + DNase-I, GSH + Cip or DNase-I + Cip) led to greater biofilm disruption and reduction in viable bacterial cells compared to a single component alone. DNase I in combination with antibacterial agents has been previously shown to increase the efficacy of antibiotics and detergents on many bacterial strains (Izano et al., 2008; Tetz et al., 2009). Considering the mechanism of action of each component of the combination treatment studied here, we conclude that GSH binds to pyocyanin to disrupt biofilm integrity, neutralizes pyocyanin cytotoxicity and also has bactericidal activity (Aquilano et al., 2014; Das et al., 2015; Kwon et al., 2015); DNase I cleaves eDNA in biofilms and consequently disrupts biofilm architecture (Whitchurch et al., 2002; Swartjes et al., 2012) and ciprofloxacin kills bacteria by inhibiting bacterial DNA replication essential for cell division, but cannot effectively penetrate the intact biofilm to kill bacteria within (Michael et al., 2010).

Our study showed that the combined GSH + DNase-I facilitates penetration of the antibiotic into the disrupted biofilm, as demonstrated by the >85% reduction in biofilm viability resulting from enhanced antibiotic activity (**Figures 2A,I**). In parallel, *P. aeruginosa* AES-1R biofilms grown in ASMDM when treated with CT, also exhibited the greatest decrease in: total biofilm biomass (ninefold) and % of biofilm area (threefold), in comparison to untreated/control biofilm (Supplementary Figure 1). Previous *in vitro* assays had shown *P. aeruginosa* biofilms grown on CF airway epithelial cells develop a dramatically heightened level of resistance to antibiotics (Yu et al., 2012). Notably, in this study we demonstrate that CT both disrupts and kills *P. aeruginosa* on lung epithelial cells without affecting the adherent cells and this factor is important to the overall efficacy of the treatment (**Figure 3**).

Pyocyanin, being a zwitterion, diffuses across the host cell membrane and undergoes redox reactions by accepting electrons from NADPH which it subsequently donates to molecular oxygen to form primarily O_2_^-^ and subsequently H_2_O_2_ through dismutation (O’Malley et al., 2003, 2004). GSH is a thiol tripeptide found in all human cells, and its intracellular level varies from 2 to 10 mM (Cheluvappa et al., 2008) and is considered to be a master antioxidant. GSH’s primary functions include defense against ROS and free radicals and maintenance of a healthy immune system. Pyocyanin-derived ROS oxidize the CF patient’s intracellular and extracellular GSH to form GSSG (21) and this decrease in GSH significantly alters host cell functions and cell death during the chronic stage of CF infection (Ran et al., 2003; O’Malley et al., 2004; Gloyne et al., 2011).

**FIGURE 9:**
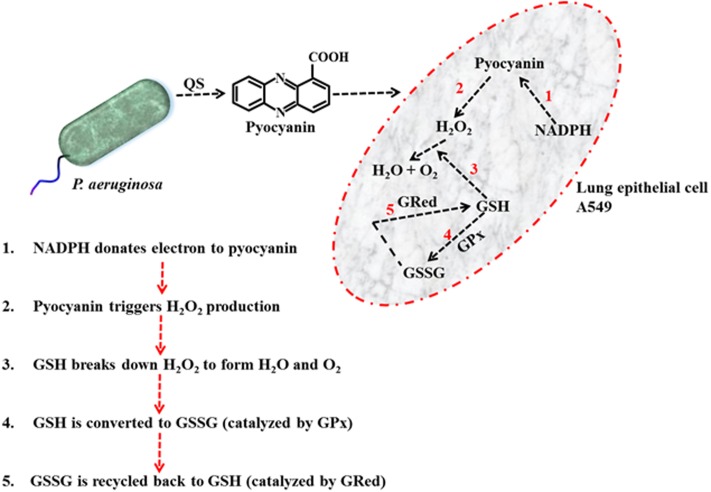
Schematic diagram illustrating the mechanism of pyocyanin mediated H_2_O_2_ production, conversion of GSH to GSSG via GPx and recycling of GSSG to GSH via GRed in A549 cell.

The results detailed in **Figure 5** provide a means to explain the pyocyanin/GPx/GRed/GSH interplay: that is, pyocyanin increases the level of ROS, and this leads to enhanced GPx activity in A549 cells because GPx catalyzes the reaction through which GSH neutralizes H_2_O_2_. In this process, GSH is converted to GSSG (Equation 1). In contrast, GRed activity is depleted in pyocyanin-treated A549 cells, thus inhibiting the recycling of GSSG to GSH; GRed is the primary factor that catalyzes the reaction essential for GSSG recycling to GSH, employing the cofactor β-NADPH_2_ as set out in Equation 1 (Aquilano et al., 2014). We also observed significant (1.9 and 1.2 times) increases in cellular GSH as a result of supplementing with exogenous GSH on pyocyanin + GSH-treated, and control + GSH-treated A549 cells, respectively. This increase could be due to two factors: (i) direct entry of exogenous GSH inside cells and (ii) GSH altering pyocyanin permeability into A549 cells. In support of the latter mechanism recent data indicate that GSH directly binds with pyocyanin through its thiol (–SH) moiety, and modulates its structure by forming GS-Pyo a cell-impermeable metabolite (Muller and Merrett, 2015). In this study, we also used GSSG as an additional control to examine whether exogenous GSSG alone enhanced intracellular GSH levels and restored the enzymatic activity and viability of A549 cells. However, our results showed that supplementation with GSSG had no impact on intracellular GSH levels (**Figure 5C**), GRed activity (**Figure 5B**) and cell viability and confluence remained unchanged (**Figure 4**). We interpret this result as being due to pyocyanin-mediated decline in GRed activity that manifests as insufficient enzymatic activity for the conversion of exogenously added GSSG to GSH. This confirms the mechanism that depletion in total GSH and increase in GSSG in pyocyanin treated A549 cells is due to inhibition of GRed activity in pyocyanin treated cells (**Figure 9**).

Depletion of intracellular GSH by added pyocyanin was previously reported by O’Malley et al. (2004) and Gloyne et al. (2011) but their study did not investigate the role of enzyme (GPx and GRed) activity in inhibiting GSH formation. In addition we also showed for the first time that supplementing GSH increases total intracellular GSH levels and this effect is directly associated with restoring A549 GRed enzymatic activity (**Figures 5B,C**). This restoration is crucial for conversion of GSSG to GSH, a mechanism that aids in the host cell defense against ROS and supports cellular function and growth (Aquilano et al., 2014). Notably, Gloyne et al. (2011) reported that pretreatment of A549 cells with synthetic antioxidant *N*-acetylcysteine enhanced GSH levels in A549 cells, however, the mechanism that underlies this increase in intracellular GSH, and whether this involved enhanced GRed enzymatic activity was not explored. We were also able to show that pyocyanin-mediated oxidative stress in A549 cells can be reduced considerably by regular (daily) treatment with GSH (**Figure 8**) and this likely facilitates the A549 cell growth seen in **Figure 7**. Other factors that trigger pyocyanin-mediated oxidative stress in airway epithelial cells include inhibition of catalase activity and depletion of NADPH (O’Malley et al., 2003; Rada et al., 2008) although this was not verified in our study.

Oxidative stress mediated by pyocyanin has a strong inhibitory effect on A549 cell multiplication (growth) and confluence. Using the IncuCyte Zoom live cell imaging system we were able to show that pyocyanin presence drastically modulated A549 cell morphology/eccentricity (**Figure 6**). Changes in cell eccentricity could be associated with cell hyperplasia and metaplasia (abnormal changes in cell or tissue morphology), which was previously reported in a study using a mouse model infected with *P. aeruginosa* (Charles et al., 2009). Deregulation in enzymatic activity and cellular function are responsible for modulation in A549 morphology, immature cell death and cell cycle arrest whereas, GSH presence with pyocyanin improved cell growth, eccentricity (**Figure 6**) and viability by scavenging ROS and free radicals directly (electron donation through the thiol moiety) or indirectly via GRed (this study) and catalase activity (O’Malley et al., 2003).

In addition to oxidative stress cytotoxicity, pyocyanin pathogenicity has shown to alter calcium homeostasis and depleting intracellular cAMP and ATP levels (Chakravarthi et al., 2006), alters human protease activity, modulates the host immune response to support evasion of the host immune system and establish chronic infection (Winstanley and Fothergill, 2008). Pyocyanin also initiates neutrophil extracellular trap (NET) formation (Rada et al., 2013) and aberrant NET release triggered by pyocyanin-mediated intracellular ROS production directly damages host tissues and has been linked to the severity of many diseases including CF (Rada et al., 2013).

The effect of GSH in neutralizing epithelial cellular cyto-toxicity has been investigated in this study. GSH efficiency in restoring various other factors includes normalizing protease levels or ATP levels, NET production and host immune response, and these are yet to be investigated. To further ascertain the translatability of the novel effects of CT as a treatment for biofilm infections will require *in vivo* study in a mammalian model of lung infection and other *in vivo* sites, and an analysis of factors linked to the host’s intrinsic immune response against bacterial infections.

## Conclusion

Bacterial biofilm infections are highly prevalent and are a significant cause of morbidity and mortality. With the rapid emergence of resistance to conventional antibiotic therapies and intrinsic biofilm resistance to antibiotic penetration, *P. aeruginosa* biofilm treatment options are limited. Consequently, novel anti-biofilm strategies to prevent biofilm formation and remove existing biofilms are being sought. The question of whether novel disruption strategies have additional benefits has not been previously explored. In addition to biofilm disruption, our study demonstrated that GSH facilitates epithelial cell re-growth and confluence by restoring intracellular GSH levels and enzymatic activity. GSH also neutralizes oxidative stress mediated by pyocyanin likely through oxidant-scavenging and rebalancing of the cellular redox status. Thus proper administration of GSH as an antimicrobial agent (either in the form of aerosol inhalation, spray, wound care gel, coatings on medical implants/equipment, oral therapy) will add a significantly to the ability to quickly treat the wide variety of *P. aeruginosa* biofilm infections, and also facilitate host tissue recovery. Another thiol-antioxidant, NAC (a precursor of GSH) has also shown great potential to disrupt multispecies endodontic biofilms, suggesting its potential use in root canal and dental plaque treatment (Suk Choi et al., 2017). Similarly vitamin C, which is also naturally found in various food sources and is a major component of dietary vitamin supplements has recently been shown to kill drug-resistant *Mycobacterium tuberculosis* (Vilcheze et al., 2013), supporting the notion that antioxidants represent a promising novel strategy to remove the biofilms of pathogenic bacteria and prevent biofilm infections in clinical settings.

## Author Contributions

TD and MS: Conducted experiments and prepared manuscript. AI: Conducted experiment. PW, MM, and JM: Prepared manuscript.

## Funding

This work was supported by the University of Sydney Postdoctoral Research Fellowship to TD and by the University of Sydney to TD and JM.

## Conflict of Interest Statement

The authors declare that the research was conducted in the absence of any commercial or financial relationships that could be construed as a potential conflict of interest.
