# Supplementary material for: Glutathione Enhances Antibiotic Efficiency and Effectiveness of DNase I in Disrupting Pseudomonas aeruginosa Biofilms While Also Inhibiting Pyocyanin Activity, Thus Facilitating Restoration of Cell Enzymatic Activity, Confluence and Viability
Source: Front Microbiol. 2017 Dec 14;8:2429. doi: 10.3389/fmicb.2017.02429 (PMC5729223; doi:10.3389/fmicb.2017.02429)
Supplement: Supplementary file 1 [file Image_1.PDF]

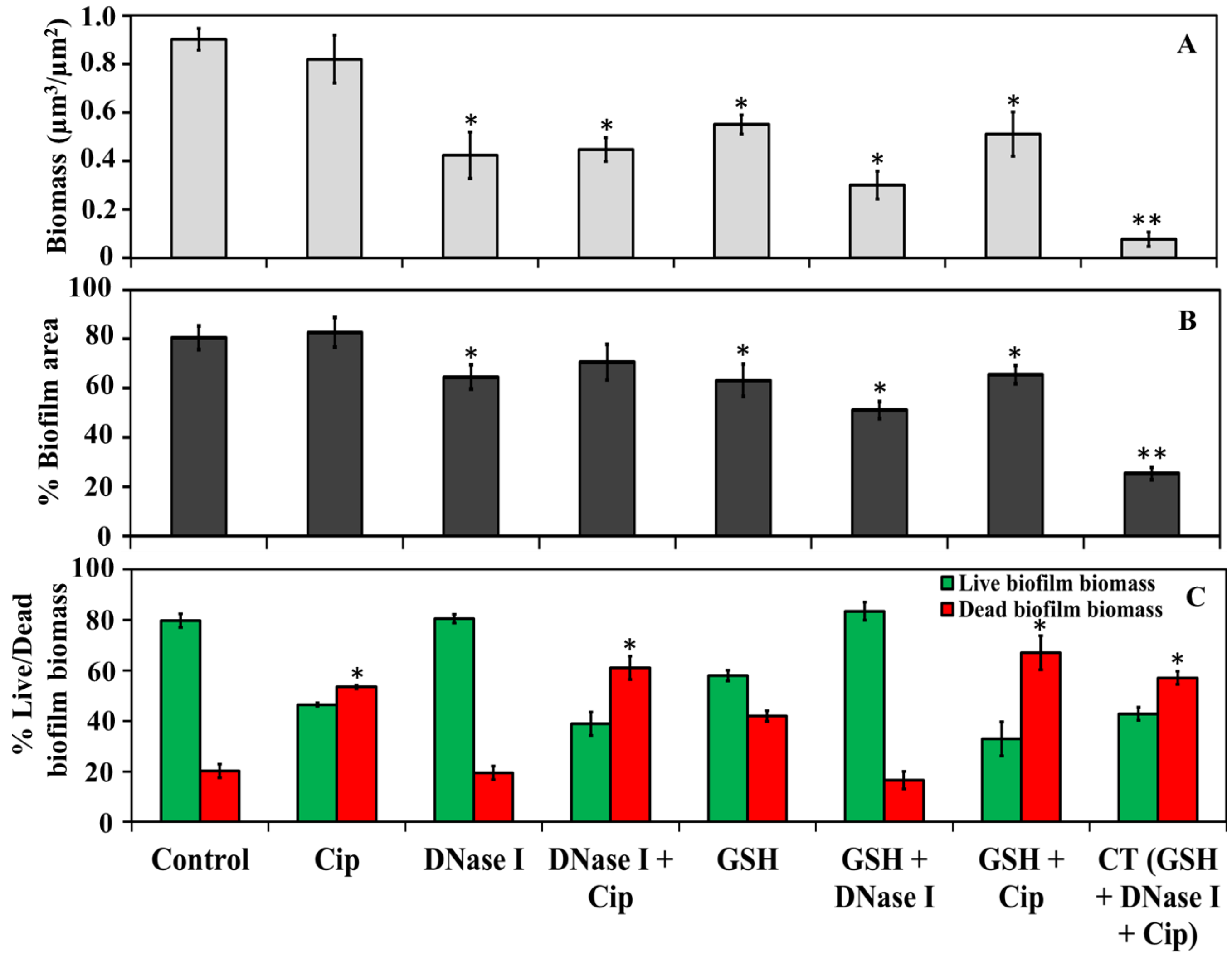

Supplementary Figure 1: Quantification of biofilm images using Image J analysis (from Figure 2 B-I) showed that when an established AES-1R biofilm (grown for 48 h) was subjected to GSH or DNase I alone, biomass decreased significantly (P < 0.05) (0.54 and 0.48μm^3/μm^2 respectively) as did the % biofilm area (62% and 64%, respectively,) when compared to untreated AES-1R (biomass = 0.89 μm^3/μm^2, % biofilm area = 80). When comparing the percentage of live *vs* dead biomass, AES-1R showed increased live biomass regardless of whether GSH (59%) or DNase I (81%) was used as treatment. In contrast, ciprofloxacin treatment significantly increased (p< 0.05) dead biofilm biomass (54%), but had no significant effect on overall biomass (0.82μm^3/μm^2) and biofilm area (81%). Combinations of GSH +

DNase I, GSH + Cip or DNase I + ciprofloxacin caused more significant biofilm disruption as measured by reduced biomass and biofilm area, than treatment with a single compound. GSH or DNase I combined with ciprofloxacin was also shown to result in a significant ($P < 0.05$) increase in dead biomass percentage (67 and 61). However, the most significant change ($P < 0.005$), was obtained when AES-1R biofilm was subjected to all three agents (GSH + DNase I + ciprofloxacin). Biomass and biofilm area value decreased significantly relative to control ($0.076 \mu m^3/\mu m^2$ and 26%, respectively) and dead biofilm was 57%. Sup. Figure 1A-B: * = $P < 0.05$ when compared to control. ** = $P < 0.005$ when compared to all treatments. Sup. Figure 1C: * = $P < 0.05$ compared to % of live biofilm cells. Results are shown as mean ± SD and all experiments were done in biological triplicate (n=3).
